# Effects of the diabetes linked *TCF7L2 *polymorphism in a representative older population

**DOI:** 10.1186/1741-7015-4-34

**Published:** 2006-12-20

**Authors:** David Melzer, Anna Murray, Alison J Hurst, Michael N Weedon, Stefania Bandinelli, Anna Maria Corsi, Luigi Ferrucci, Guiseppe Paolisso, Jack M Guralnik, Timothy M Frayling

**Affiliations:** 1 Peninsula Medical School, RD&E Wonford Site, Barrack Road, Exeter, EX2 5DW, UK; 2 Laboratory of Clinical Epidemiology, Italian National Research Council on Aging, Geriatrics Department, Florence, Italy; 3 Tuscany Regional Health Agency, Firenze, Italy, I.O.T. and Department of Medical and Surgical Critical Care, University of Florence, Italy; 4 Longitudinal Studies Section, Clinical Research Branch, Gerontology Research Center, National Institute on Aging, Baltimore, Maryland, USA; 5 Department of Geriatric Medicine and Metabolic Disease, University of Naples, Naples, Italy; 6 Laboratory of Epidemiology, Demography and Biometry, National Institute on Aging, Bethesda, Maryland, USA

## Abstract

**Background:**

A polymorphism in the *transcription factor 7-like 2 *(*TCF7L2*) gene has been found to be associated with type 2 diabetes in case-control studies. We aimed to estimate associations of the marker rs7903146 (C/T) polymorphism with fasting glucose, lipids, diabetes prevalence and complications in an older general population.

**Methods:**

In total, 944 subjects aged ≥ 65 years from the population representative InCHIANTI study were enrolled in this study. Those with fasting blood glucose of ≥ 7 mmol/l or physician diagnosis were considered diabetic. Cut-off points for impaired fasting glucose (IFG) were ≥ 5.6 mmol/l to < 7 mmol/l.

**Results:**

In the general population sample, minor (T) allele carriers of rs7903146 had higher fasting blood glucose (FBG) (*p *= 0.028) but lower fasting insulin (*p *= 0.030) and HOMA2b scores (*p *= 0.001), suggesting poorer beta-cell function. T allele carriers also had smaller waist circumference (*p *= 0.009), lower triglyceride levels (*p *= 0.006), and higher high-density lipoprotein cholesterol (*p *= 0.008).

The prevalence of diabetes or IFG was 32.4% in TT carriers and 23.3% in CC carriers; adjusted OR = 1.67 (95% confidence interval 1.05 to 2.65, *p *= 0.031). Within the diabetic and IFG groups, fewer T allele carriers had metabolic syndrome features (*p *= 0.047) or had experienced a myocardial infarction (*p *= 0.037). Conversely, T allele carriers with diabetes had poorer renal function (reduced 24-hour creatinine clearance, *p *= 0.013), and possibly more retinopathy (*p *= 0.067). Physician-diagnosed dementia was more common in the T carriers (in diabetes *p *= 0.05, with IFG *p *= 0.024).

**Conclusion:**

The *TCF7L2 *rs7903146 polymorphism is associated with lower insulin levels, smaller waist circumference, and lower risk lipid profiles in the general elderly population. Patients with diabetes who are carriers of the minor allele are less likely to have metabolic-syndrome features, but may experience more microvascular complications, although the number of cases was small. If replicated, these findings may have implications for developing treatment approaches tailored by genotype.

## Introduction

Type 2 diabetes mellitus is an important disabling condition[1], and its prevalence is increasing. Type 2 diabetes mellitus is generally understood to be a metabolic disorder defined by hyperglycemia and insulin resistance combined with abdominal obesity, dyslipidemia and hypertension. The current focus of management of diabetes mellitus has moved from controlling hyperglycemia alone to a multifactorial approach to risk reduction[2,3].

An association has recently been established between type 2 diabetes and variation in the transcription factor *TCF7L2 *gene in a large Icelandic study[4]. Two additional cohorts showed similar associations, giving a combined odds ratio (OR) = 1.56, 95% confidence interval (CI) 1.41 to 1.73, *p *= 4.7 × 10^-18 ^per allele. The rs7903146 single nucleotide polymorphism (SNP) in *TCF7L2 *is in linkage disequilibrium with the risk microsatellite, and has been recommended for use in replication studies[4]. In a separate study of progression to diabetes in the Diabetes Prevention Program, Florez et al[5] found that the same variant in *TCF7L2 *was associated with an increased risk of diabetes incidence among people with impaired glucose tolerance. Several case-control studies have now replicated these findings [6-11].

Thus far, the available evidence for *TCF7L2 *has been from case-control comparisons or intervention trial volunteers, and not representative general populations; data from elderly populations, in whom risks of type 2 diabetes are highest, are scarce. As Sackett and Haynes[12] have pointed out, many biomarkers have appeared promising in comparisons of clear-cut cases and controls, only to perform poorly in the general population. In maturity onset diabetes of the young, the clinical presentation and treatment needs of patients varies markedly by genotype[13]; whether such clinical heterogeneity by genotype is present for *TCF7L2 *and type 2 diabetes is unclear.

Our study aimed to investigate whether the presentation of diabetes and related features is associated with *TCF7L2 *genotype in a representative population of older people. We genotyped the *TCF7L2 *SNP rs7903146, and correlated genotype with diabetes-related biochemical changes and measures of diabetes complications.

## Methods

The data were from the InCHIANTI Study[14] of aging, a representative sample of 1155 people aged ≥ 65 years in two towns in Tuscany, Italy. Subjects had an initial assessment at home followed by two clinic visits between September 1998 and March 2000. The participants were all of white European origin. The Italian National Institute of Research and Care of Aging Institutional Review Board approved the study.

The InCHIANTI cohort has been studied extensively for phenotypic measures of aging, including biochemical and physical tests. We analyzed data on fasting plasma glucose and insulin levels, and calculated Homeostasis Model Assessment (HOMA2) scores using a formula from the Oxford Centre for Diabetes, Endocrinology and Metabolism (, accessed June 2006). Fasting insulin was determined using a commercial double-antibody, solid-phase radioimmunoassay (Sorin Biomedica, Milan, Italy) with an intra-assay coefficient of 3.1%[15]. Fasting blood glucose was determined by an enzymatic colorimetric assay using a modified glucose oxidase-peroxidase method (Roche Diagnostics GmbH, Mannheim, Germany) and a Roche-Hitachi 917 analyzer.

Diseases were ascertained by an experienced clinician using pre-established criteria combining self-reported physician diagnoses, current pharmacologic treatment, medical records, clinical examinations, and blood tests[16]. The "diabetes" group in this analysis had fasting blood glucose levels of ≥ 7.0 mmol/l or a physician diagnosis of diabetes with diabetes diet or drug treatment in the previous 2 weeks. Impaired fasting glucose was defined according to American Diabetes Association (ADA) criteria[17,18] as a fasting glucose of 5.6–6.9 mmol/L, excluding cases diagnosed as diabetic.

A 24-hour urine collection was performed, and urinary and serum creatinine were measured using a modified Jaffe method. A creatinine clearance of ≤ 30 mL/min was considered renal insufficiency, a threshold previously shown to be associated with anemia in older people in this dataset[19]. For metabolic syndrome, measures were dichotomized in accordance with the Adult Treatment Panel III (ATP III) definitional criteria[20]: men are classified ashaving abdominal obesity if their waist circumference is > 1020 mm, women if their waist circumferenceis > 880 mm. For both sexes a high triglyceride level was considered to be ≥ 150 mg/dL. Men were classified as having low high-density lipoprotein (HDL) levels if the serum level was ≤ 40 mg/dL; for women the cut-point was ≤ 50 mg/dL.

### Genotyping

The rs7903146 SNP was genotyped using a *Taq*Man PCR assay: 20 ng of genomic DNA was amplified in each assay in the presence of specific probes for each SNP variant labeled with fluorescent dye at the 5' end and a quencher molecule at the 3' end (designed by Applied Biosystems' "assays-by-design" service). PCR was carried out in 1 μl reaction volumes with 1× ABsolute™QPCR mix (ABgene) and 1× probe mix (Applied Biosystems). An initial denaturation at 95°C for 10 minutes was followed by 40 cycles of PCR, with 15 seconds denaturation at 95°C, and 1 minute annealing/extension at 60°C. Following 40 cycles of PCR, fluorescence was measured for each probe on a Pherastar plate reader and compared with an internal control ROX dye standard. Genotypes were assigned using Klustercaller software (Kbioscience™). A percentage (10%) of samples was duplicated and scored blind.

### Statistics

Linear regression models used log-transformed values of all serum levels, adjusted for age and sex. The distribution of insulin and insulin^2 ^(in pmol/mL) were not normally distributed on skewness and kurtosis normality testing (*p *< 0.0001 for both). Transforming to log(insulin) and log(insulin^2^) yielded normality test *p *values of 0.0085 and 0.6438 respectively; we therefore used log(insulin^2^) in linear models, which assume normal distributions.

Logistic regression models were used for the age- and sex-adjusted categorical associations. Type 2 diabetes is more common in men, but in the adjusted models, sex did not reach significance, and therefore all analysis was performed with both sexes together.

## Results

### Characteristics of subjects

There were 944 subjects for whom DNA available and genotyped for *TCF7L2 *(Table 1) aged ≥ 65 years. In this general population sample, the mean age was 75 years (range 65–102). The proportion in the obese category (BMI ≥ 30) was 32% in the whole sample and 41% in the group with diabetes. Similar differences were evident on abdominal obesity (with sex-specific waist measurements).

The frequencies of each genotype in the whole sample were similar to published data (Table 1). There were no inconsistencies among the duplicate samples and no significant deviation from Hardy-Weinberg equilibrium (σ^2 ^= 0.012, *p *= 0.912).

### Effect of *TCF7L2 *in the general elderly population sample

In the general elderly population sample, there was a significant trend toward higher fasting glucose levels with increasing number of T alleles (mean fasting glucose 5.31 mmol/L in the TT group, 5.19 mmol/L in the CT group, and 5.08 mmol/L in the CC group; age and sex adjusted per allele regression model *p *= 0.028) (Table 2). Logged fasting insulin^2 ^levels were significantly lower in the T allele groups and there was also a strong trend per T allele of reduction in HOMA2b measures, suggesting impaired beta-cell function (96% in TT versus 113% in CC group, regression *p *= 0.001). HOMA2S-measured insulin sensitivity increased with increasing number of T alleles but this did not reach formal significance. However, upon removing patients who were on drug treatment, the association with HOMA2S was significant (β = 0.057, 95% CI 0.006 to 0.108, *p *= 0.027).

In addition to the glucose/insulin axis changes, there were also changes in lipid levels, with the T allele being associated with significantly higher levels of HDL cholesterol (*p *= 0.008 in the per allele model) and lower levels of triglycerides (*p *= 0.006 in per allele model), suggesting a lower risk lipid profile. These associations were independent of the marginal association with insulin sensitivity: on adjusting for logged HOMA2ir values, both the triglyceride (per allele β = -0.0568, 95% CI -0.101 to -0.0125, *p *= 0.012) and HDL associations (per allele β = 0.030, 95% CI 0.003 to 0.053, *p *= 0.027) remained.

When subjects with diabetes or impaired fasting glucose were excluded from the models in Table 2, serum levels of HDL and triglycerides remained associated with the rs7903146 genotype (per allele β = 0.037, 95% CI 0.009 to 0.066, *p *= 0.010 and β = -0.058, 95% CI -0.107 to -0.009, *p *= 0.021 respectively). None of the other measures in Table 2 was significant after removal of the diabetic and impaired fasting glucose (IFG) groups.

The waist circumference of those with the rs7903146 T allele was significantly smaller (β = -0.0148, 95% CI -0.0259 to 0.0037, *p *= 0.009). This association was little changed by excluding those with diabetes or IFG (age and sex adjusted per allele β = -0.0209, 95% CI -0.0334 to -0.008, *p *= 0.001).

### Characteristics of those with diabetes

The prevalence of diabetes was 12.4% in the CC group and 18.9% in the TT group (Table 3); when combined with ADA criteria-based IFG, the prevalences were 23.3% and 32.4% respectively (per minor allele risk OR = 1.29, 95% CI 1.04 to 1.60, *p *= 0.023). There was a non-significant trend to higher risks in the heterozygous group, possibly due to the limited sample size.

We next examined whether the characteristics of those with diabetes varied by *TCF7L2 *status (Table 4). The TC and TT groups were combined, owing to the small numbers. The T allele carrier patients with diabetes and the wider group of patients with diabetes plus IFG were significantly less likely to have two or more ATP III-based features[20] of metabolic syndrome (excluding hyperglycemia), thus diabetic T allele carriers had an OR = 0.37 (95% CI 0.15 to 0.92, *p *= 0.033) for metabolic syndrome as defined by the presence of hypertension, abdominal obesity, high triglycerides and low HDL levels. This included T allele carriers being markedly less likely to have hypertension (OR-0.37, 95% CI 0.16 to 0.90, *p *= 0.028). Treating the relevant measures as continuous variables showed the same pattern of associations.

There was no difference in the length of time that patients had had diabetes (13.1 years in the CC group, 12.1 years in the T carrier (CT/TT) group, *p *= 0.68). There was also no significant association with treatment for diabetes: 59.2% in the CC group and 66.7% in the T group were on some form of treatment (*p *= 0.393), although there was a trend towards T allele carriers requiring more drug treatment (*p *= 0.091) (60.1% on oral hypoglycemics or insulin versus 44.9% in the CC group).

Numbers with complications were small (Table 4). However, we found suggestive evidence that T allele carriers are less likely to have had a myocardial infarction, a key macrovascular complication (for T carrier patients with diabetes OR = 0.16, 95% CI 0.03 to 0.84, *p *= 0.030; for T carrier patients with diabetes plus IFG OR = 0.27, 95% CI 0.08 to 0.92, *p *= 0.037). Conversely, T allele carriers were more likely to have the key microvascular complication of poor renal function, measured by 24-hour creatinine clearance (T carrier patients with diabetes OR = 3.15, 95% CI 1.27 to 7.81, *p *= 0.013); this was also significant in linear models treating creatinine clearance as a continuous variable (diabetes, β = -10.22, 95% CI -18.93 to -1.52, *p *= 0.022; and combined β = -5.99, 95% CI -11.92 to -0.06, *p *= 0.048). Eleven of the 12 cases of retinopathy occurred in the T carriers (CT/TT group) with diabetes (*p *= 0.067). A further interesting finding was that 13 of the 14 cases of physician-diagnosed dementia in the diabetes plus IFG group were T allele carriers (*p *= 0.024), including all 10 cases of vascular dementia (chi^2 ^*p *= 0.014).

## Discussion

The *TCF7L2 *(rs7903146) polymorphism is the most powerful single genetic variant influencing type 2 diabetes risks identified to date[21]. In this study, we aimed to explore the effects of this polymorphism in a general elderly population, rather than in selected cases and controls. We found that the *TCF7L2 *polymorphism is associated with higher fasting glucose and lower insulin and HOMA2b levels across this sample, suggesting beta-cell impairment. Unexpectedly, however, we also found that the rarer allele (T) is also associated with a more protective lipid profile of higher HDL levels and lower triglyceride levels. This latter finding was still significant after adjusting for insulin resistance, suggesting that it is not secondary to the possible increase in insulin sensitivity that we observed. The improved lipid profile was present even in the population without diabetes or IFG (in whom a smaller waist circumference was also found) and therefore this finding is unlikely to be an artifact of *TCF7L2 *polymorphism carriers developing diabetes at lower levels of abdominal obesity.

We also found that within the group of patients with diabetes and IFG, T allele carriers were less likely to have metabolic syndrome features (*p *= 0.047) including hypertension, or to have had a myocardial infarction (*p *= 0.037). This suggests a lower macrovascular risk factor loading in the T allele group compared with the common CC genotype. In contrast to the lower lipid risk profile, however, T allele carriers with diabetes and IFG were more likely to have certain microvascular-related complications, including poor renal function and perhaps a higher rate of retinopathy. The association with physician diagnoses of dementia in the T carriers with diabetes and IFG was also unexpected, but may fit into the profile of raised microvascular risk factors. The existence of an association between dementia and diabetes was supported by a recent systematic review[22].

The incidence of hypertension, macrovascular disease and type 2 diabetes all increase with age[23], and prior to the discovery of *TCF7L2 *it was suggested that a common mechanism might be responsible for all of these pathological states, as these conditions appear to cluster in the same individuals. Patients with diabetes mellitus have a much higher rate of cardiovascular disease (CVD) than the general population, and dyslipidemia and other metabolic syndrome features seen in diabetes are accepted as the major risk factors for CVD[24,25]. Clinical trials have confirmed that lipid-lowering therapies result in a substantial reduction of cardiovascular risk in patients with type 2 diabetes[26]. Our finding of lower rates of myocardial infarction, although based on small numbers, is consistent with patients with diabetes who carry the T allele having a lower risk profile of metabolic-syndrome features.

In contrast to macrovascular changes, diabetic microvascular complications (including retinopathy[27], neuropathy and nephropathy[28]) are mainly linked to hyperglycemia[29]. Intensive blood-glucose control decreases the risk of microvascular complications, but not macrovascular disease, in patients with type 2 diabetes[30]. Our finding of higher rates of microvascular complications in T allele patients with diabetes is consistent with specific dysfunction of insulin production in the beta cell present with the *TCF7L2 *polymorphism[4], perhaps leading to greater exposure to hyperglycemia-mediated damage. The prevailing biochemical theories on how diabetes leads to microvascular disease[31] includes several glucose-related pathways that contribute to endothelial damage and dysfunction.

In evaluating these results, it is important to note that the data are cross-sectional, and could perhaps have been affected by differential survival of high-risk participants. In theory, the lower risk lipid profile of T allele carriers may be due to a markedly higher mortality rate in T carriers who had had metabolic syndrome features plus the T allele-associated diabetes risk, and therefore did not survive past 65 years of age. However, there was no significant association between T allele status and age in this study (linear sex adjusted regression β = 0.031, *p *= 0.963) and there was also less dyslipidemia in the general population, excluding those with diabetes or IFG, making a differential mortality scenario less likely. A second limitation is our relatively crude measure of beta-cell function; more accurate measurements are obtained from oral glucose tolerance tests, but these were not performed in InCHIANTI. In addition, the numbers of patients with diabetes with major complications are limited, and although the findings presented are consistent with previous knowledge of the mechanisms of diabetes-related complications, replication of these findings in independent populations is clearly needed. Two-thirds of those with diabetes were receiving some form of treatment, and prior treatment may have influenced diabetes-specific estimates, although rates of treatment were not significantly different by SNP status. As treated patients with diabetes are likely to have more severe disease and complications than untreated patients, simple exclusion or adjustment for treatment would be misleading. The presence of many of the associations in the general population sample and the combined diabetes and IFG groups, in which the effect of treatment would be diluted, suggests that these findings are robust.

If these findings are confirmed in other populations and in prospective cohort studies, the presentation of T allele carriers as having lower risk of metabolic syndrome but greater risk of microvascular complications would have obvious clinical implications. The desegregation of type 2 diabetes into subtypes with different patterns of risk suggests that treatment approaches, especially to lipid reduction and closeness of glycemic control, could be more closely tailored to genetic subtype.

## Conclusion

The *TCF7L2 *polymorphism is associated with beta-cell dysfunction but also a more protective lipid risk profile in the general elderly population. *TCF7L2 *minor allele carrier patients with diabetes are less likely to have metabolic syndrome features, but may be at higher risk from more microvascular complications, including renal impairment and (vascular) dementia. If replicated, these findings may have implications for developing treatment approaches tailored to the balance of risks in the different genotype groups.

## Competing interests

The author(s) declare that they have no competing interests.

## Authors' contributions

DM contributed to the design of this study, and led the statistical analysis, writing and editing of the manuscript. AM participated in the co-ordination and the design of the study, undertook the genotyping and contributed to the editing of the manuscript.

AJH assisted with the statistical analysis and editing of the manuscript. MNW undertook the analysis of the genotyping for quality control. SB, LF and JG lead the InCHIANTI study including design and data collection, and contributed to the editing of the manuscript. AMC prepared the InCHIANTI DNA and contributed to the editing of the manuscript. GP carried out the insulin assays, and contributed to the editing of the manuscript. TMF contributed to the study design and genotyping, and contributed to the editing of the manuscript. All authors read and approved the final manuscript.

## Pre-publication history

The pre-publication history for this paper can be accessed here:



## Figures and Tables

**Table 1:** Characteristics of the InCHIANTI study general population sample aged ≥ 65 years, and the subgroup with diabetes

	All subjects ≥ 65 years	Subjects with diabetes
Number of subjects	944	127
Male	415 (44%)	64 (50%)
Age (years)	75 ± 7.4	74 ± 6.8
BMI		
< 25 kg/m^2^	251 (27%)	23 (18%)
25–29 kg/m^2^	395 (42%)	52 (41%)
30+ kg/m^2^	298 (32%)	52 (41%)
Subjects with abdominal obesity*	343 (40%)	60 (47%)
Fasting glucose levels		
< 6 mmol/L	774 (82%)	31 (24%)
6–6.9 mmol/L	70 (7%)	18 (14%)
7+ mmol/L	100 (11%)	78 (61%)
Insulin levels (pmol/L)	68.6 ± 1.7	76.3 ± 1.7
HOMA beta cell function (%)	107.7 ± 1.6	59.6 ± 1.9
HOMA insulin sensitivity (%)	75.9 ± 1.6	62.7 ± 1.6
Total cholesterol (mg/dL)	213.1 ± 1.2	205.3 ± 1.2
LDL (mg/dL)	131.2 ± 1.3	124.7 ± 1.3
HDL (mg/dL)	53.7 ± 1.3	49.2 ± 1.3
Triglycerides (mg/dL)	114.1 ± 1.6	124.7 ± 1.7
rs7903146 genotypes		
CC	394 (42%)	49 (39%)
CT	439 (47%)	57 (45%)
TT	111 (12%)	21 (17%)

* Men were classified as being abdominally obese if their waist circumference was more than 1020 mcm, women if their waist circumference was more than 880 mm.

Data are *n *(%) or mean ± SD.

**Table 2:** Serum levels by genotype in the whole InCHIANTI population aged ≥ 65 years, with age- and sex-adjusted linear regression model estimates of associations with logs of measures

					Model: age and sex adjusted, for log of measure		
					
		*n*	Mean	SD	Beta	SE	p value
Glucose (mmol/L)	CC	379	5.08	1.22	0		
	CT	432	5.19	1.23	0.024	0.015	0.100
	TT	109	5.31	1.27	0.044	0.022	0.052
			Per minor allele		0.022	0.010	0.028
Insulin^2^	CC	364	81.74	1.69	0		
pmol	CT	416	78.87	1.70	-0.038	0.035	0.276
	TT	103	70.48	1.55	-0.121	0.054	0.026
			Per minor allele		-0.053	0.025	0.030
HOMA beta cell function (%)	CC	362	113.19	1.57	0		
	CT	415	106.23	1.61	-0.067	0.034	0.049
	TT	103	95.74	1.69	-0.166	0.052	0.002
			Per minor allele		-0.078	0.024	0.001
HOMA insulin sensitivity (%)	CC	362	74.06	1.63	0		
	CT	415	76.68	1.66	0.032	0.035	0.362
	TT	103	82.80	1.55	0.110	0.054	0.043
			Per minor allele		0.048	0.025	0.053
Cholesterol total mg/dL	CC	379	212.92	1.21	0		
	CT	432	214.35	1.20	0.007	0.013	0.605
	TT	109	208.83	1.21	-0.017	0.020	0.399
			Per minor allele		-0.004	0.009	0.680
LDL mg/dL	CC	379	131.38	1.31	0		
	CT	432	132.27	1.29	0.008	0.018	0.670
	TT	109	126.73	1.33	-0.033	0.028	0.251
			Per minor allele		-0.009	0.013	0.495
HDL mg/dL	CC	379	52.55	1.29	0		
	CT	432	54.13	1.31	0.026	0.018	0.141
	TT	109	56.32	1.30	0.072	0.027	0.009
			Per minor allele		0.033	0.012	0.008
Triglycerides mg/dL	CC	379	118.24	1.57	0		
	CT	432	113.5	1.58	-0.040	0.032	0.212
	TT	109	102.65	1.56	-0.141	0.049	0.004
			Per minor allele		-0.061	0.022	0.006
BMI kg/m^2^	CC	356	27.75	4.06	0		
	CT	402	27.18	4.16	-0.532	0.292	0.069
	TT	101	27.37	3.56	-0.347	0.451	0.442
			Per minor allele		-0.285	0.205	0.165
Waist Circumference cm	CC	358	93.79	9.88	0		
	CT	409	91.93	10.66	-0.018	0.008	0.021
	TT	99	91.35	10.24	-0.026	0.012	0.036
			Per minor allele		-0.015	0.006	0.009

**Table 3:** Prevalence of diabetes and impaired fasting glucose, plus logistic regression odds ratios by genotype

					Age- and sex-adjusted ordinal logistic model		
					
Condition	Genotype	Not case	Case	%	OR	95% CI	p value
Diabetes	CC	345	49	12.44	1.00		
	CT	382	57	12.98	1.06	0.70–1.60	0.774
	TT	90	21	18.92	1.64	0.93–2.87	0.085
	Per minor allele				1.17	0.80–1.72	0.415
Impaired fasting glucose – ADA criteria (diabetics excluded)	CC	355	39	9.90	1.00		
	CT	379	60	13.67	1.45	0.94–2.23	0.089
	TT	96	15	13.51	1.42	0.75–2.69	0.279
	Per minor allele				1.25	0.94–1.67	0.126
Diabetes or IFG (ADA)	CC	306	88	23.34	1.00		
	CT	322	117	26.65	1.28	0.93–1.76	0.132
	TT	75	36	32.43	1.67	1.05–2.65	0.031
	Per minor allele				1.29	1.04–1.60	0.023

**Table 4:** Characteristics of patients with diabetes and ADA diagnosed hyperglycemia patients by *TCF7L2 *genotype

		Diabetes					Diabetes and impaired fasting glucose				
		
	Genotype	*n*	%	Age- and sex-adjusted OR	95% CI	p value	*n*	%	Age- and sex-adjusted OR	95%CI	p value
Two or more metabolic syndrome features	CC	33	73.3				56	66.7			
	CT/TT	41	58.6	0.37	0.15–0.92	0.033	78	55.7	0.55	0.30–0.99	0.047
High blood pressure or anti-hypertension drugs	CC	37	78.7				64	74.4			
	CT/TT	47	61.8	0.37	0.16–0.90	0.028	101	67.8	0.65	0.35–1.20	0.170
Abdominal obesity	CC	25	54.4				44	51.8			
	CT/TT	35	49.3	0.53	0.20–1.35	0.180	67	46.9	0.67	0.36–1.22	0.188
High triglycerides	CC	20	41.7				39	44.8			
	CT/TT	25	32.5	0.68	0.32–1.43	0.307	49	32.2	0.58	0.33–0.99	0.047
Low HDL levels	CC	17	35.4				31	35.6			
	CT/TT	26	33.8	0.83	0.37–1.86	0.651	44	29	0.67	0.37–1.21	0.185
Myocardial infarction	CC	7	14.6				8	9.2			
	CT/TT	2	2.6	0.16	0.03–0.84	0.030	4	2.6	0.27	0.08–0.92	0.037
Poor renal function (24-hour creatinine clearance)	CC	12	26.1				31	36.9			
	CT/TT	32	47.1	3.15	1.27–7.81	0.013	69	49.3	1.74	0.96–3.17	0.066
Retinopathy reported	CC	1	2.0				1	1.1			
	CT/TT	11	14.1	7.15	0.87–58.51	0.067	11	7.2	6.13	0.77–48.86	0.087
Dementia (physician diagnosed)	CC	1	2.0				1	1.1			
	CT/TT	8	10.3	10.67	1.00–113.70	0.050	13	8.5	11.62	1.38–97.57	0.024

HDL, high-density lipoprotein.
